# Utilization of Foamed Glass as an Effective Adsorbent for Methylene Blue: Insights into Physicochemical Properties and Theoretical Treatment

**DOI:** 10.3390/ma16041412

**Published:** 2023-02-08

**Authors:** Hussein Al-kroom, Hamdy A. Abdel-Gawwad, Mohamed Abd Elrahman, Saleh Abdel-Aleem, Mohamed Saad Ahmed, Yasser F. Salama, Saleh Qaysi, Mateusz Techman, Moaaz K. Seliem, Osama Youssf

**Affiliations:** 1 Department of Civil Engineering, School of Engineering, The University of Jordan, Amman 11942, Jordan; 2 Housing and Building National Research Center (HBRC), Raw Building Materials and Processing Technology Research Institute, Cairo 12311, Egypt; 3 Structural Engineering Department, Mansoura University, Mansoura 35516, Egypt; 4 Chemistry Department, Faculty of Science, Fayoum University, Fayoum 63514, Egypt; 5 Geology and Geophysics Department, College of Science, King Saud University, Riyadh 11362, Saudi Arabia; 6 Geology Department, Faculty of Science, Beni-Suef University, Beni-Suef 62521, Egypt; 7 Faculty of Civil and Environmental Engineering, West Pomeranian University of Technology in Szczecin, 70310 Szczecin, Poland; 8 Faculty of Earth Science, Beni-Suef University, Beni-Suef 62521, Egypt

**Keywords:** glass waste, foamed glass, alkali activation, porous structure, basic dyes, statistical modeling, mechanism

## Abstract

This study reports a potential approach for the valorization of glass waste (GW) that is mainly composed of amorphous silica to prepare lightweight foamed glass (FG). The preparation of FG was achieved by mixing sodium hydroxide with GW powder followed by sintering at a temperature of 800 °C. As-synthesized FG was characterized and applied as an effective adsorbent for the removal of hazardous organic water contaminants, in particular, methylene blue (MB) dye. FG exhibited porosity of 91%, bulk density of 0.65 g/cm^3^, compressive strength of 4 MPa, and thermal conductivity of 0.27 W/m·K. Theoretical treatment indicated that a monolayer model with one energy site was the best in fitting the removal of MB molecules. The number of MB molecules per active site (n) ranged from 2.20 to 1.70, suggesting vertical orientation and a multi-molecular adsorption mechanism. The density of FG receptor sites (D_M_) increased with the temperature, and this parameter played a vital role in the adsorption process. The adsorption capacity (Q_sat_) increased from 255.11 to 305.58 mg/g, which signifies endothermic interactions. MB adsorption on FG was controlled by physical forces such as electrostatic interactions (i.e., the adsorption energies were <20 kJ/mol). The results of this study prove the feasibility of glass waste as an effective and low-cost adsorbent for water remediation.

## 1. Introduction

The production processes of numerous products, such as silk, wool, leather, silk, and paper, generate huge quantities of waste dyes. The continuous release of these water-soluble compounds into water bodies is tremendously harmful to the environment and is considered hazardous for human beings [1,2,3]. In particular, exposure to methylene blue (MB; the tested cationic dye) can cause vomiting, cyanosis, and cancer [4,5]. MB is considered highly soluble (40 g/L) and stable [6]. Different techniques, including coagulation, advanced oxidation, biological treatment, and adsorption, can be used to remove MB from water [7,8]. The literature review has shown that the adsorption technique is particularly effective, simple, and low cost, making it highly applicable in water remediation [9,10,11].

In the majority of the former studies, the Langmuir and Freundlich equations were used as common traditional equilibrium models to evaluate the adsorption process [2,3]. However, these classical adsorption models have no connection with the steric and energetic factors governing the removal process on the molecular scale [5]. Moreover, the suggestions of Langmuir and Freundlich models are inadequate to describe the horizontal or vertical geometry of the adsorbed chemical species [3]. On the contrary, the application of advanced statistical physics models can offer new insights into the adsorption processes via the calculation of the number of MB molecules that can be removed by the FG adsorbent active sites (n), the density of FG adsorption sites (D_M_), the maximum adsorption capacity at saturation (Q_sat_), and the corresponding adsorption energies (ΔE). Thus, the theoretical treatment based on the statistical physics theory was recommended to be an effective approach to find the relation between the adsorption equilibrium and the physicochemical parameters [5]. These steric and energetic parameters allow one to better understand the removed MB geometry and mechanism on the molecular scale [12,13].

The reuse of industrial solid wastes in the design of new materials with thermal and chemical stability is recommended to be a safe method for the potential disposal of these solid wastes [14,15,16,17]. The alkali activation process is considered one of the main anticipated techniques and has a high value in the sustainable consumption of solid wastes. In recent years, foamed materials have gained increased attention in water remediation due to their high thermal stability and porous structure [14,15,16,17,18,19,20,21,22]. Numerous studied focused on utilizing alkali-activated foamed materials for the removal of contaminants from water [23,24,25]. Foamed materials produced from alkali-activated fly ash [26], volcanic tuffs [27], and hypergolic coal gangue were utilized in the removal of water contaminants. Moreover, a 3D-printed material-based glass waste (GW) was employed as an adsorbent for the uptake of dye from contaminated water [28,29]. In addition, waste-derived glass was utilized as a precursor in the fabrication of highly porous foams to remove dye from solutions under UV irradiation [30].

In the present work, GW was recycled to produce foamed glass (FG) through a simple production process. The mechanical and physical properties of the developed FG (e.g., compressive strength, bulk density, porosity, and thermal conductivity) were determined. Numerous techniques (e.g., XRD, FTIR, and FESEM) were used to study the phase composition and the morphological features of FG. The produced FG was tested as an adsorbent for the uptake of methylene blue (MB) dye from solutions. Statistical physics models (i.e., monolayer, double-layer, and multilayer) were applied to the fitting of the experimental data at 25 °C, 40 °C, and 50 °C. The steric and energetic parameters from the advanced statistical physics models were used to clarify the efficiency of FG in MB dye removal and offer new insights into the adsorption mechanism considering the molecular level. Overall, the present study suggests a sustainable method that uses the silica-rich solid wastes for the production of effective materials used for treating MB-containing solutions.

## 2. Materials and Methods

### 2.1. Materials

Glass waste (GW) was used as a main precursor for preparing foamed material. GW was supplied from Sphinx Company for Glass Manufacturing (Giza, Egypt). Ultra-pure sodium hydroxide (NaOH), which was purchased from LOBA Chemical Company (Mumbai, India), was used as fluxing material. Table 1 shows the chemical composition of GW, as determined using X-ray fluorescence (XRF). GW foam (GWF) was synthesized by exposing the GW and NaOH mixture to elevated temperature to induce GW sintering, softening, and bloating [20]. Several trials were conducted to determine the optimal conditions and to acquire optimal the foaming process. NaOH was mixed with GW in a weight ratio of 0.02. The water-to-GW ratio was set to 0.17. The foaming process was performed under the following conditions: 2% NaOH (wt% of GW), 50 kN compaction force, 800 °C with heating rate of 20 °C/min. and retention time of 2 h.

### 2.2. Methods

The mechanical and physical properties of FG were determined by measuring compressive strength, bulk density, porosity, and thermal conductivity. All tests were conducted on three cubes with dimensions of 20 mm × 20 mm × 20 mm. A compressive strength test was conducted using German-Bruf-Pressing Machine with a maximum capacity of 175 kN. The test was conducted in accordance with [31]. Total porosity was measured with mercury intrusion data using an Auto Pore IV 9500 porosimeter (Micromeritics Instrument Corporation, Norcross, GA, USA). Bulk density was determined by dividing the dry weight of the foamed sample by its volume. The mineralogical composition of GWF was determined with X-ray diffraction (XRD) using a Philips PW3050/60 diffractometer (Philips, Amsterdam, The Netherlands). It was performed in the 2θ range of 5° to 50°, at a scanning rate of 1 s/step and resolution of 0.03°/step. The functional groups inside GF were identified via Fourier transform infrared (FT-IR) spectroscopy using a KBr discussing Genesis-IIFT-IR spectrometer in the range of 400–4000 cm^−1^. The internal pore system of FG was characterized with field emission scanning electron microscopy (FESEM). The thermal conductivity of FG was measured using a KD2-Pro handheld device (Decagon Devices Inc., Pullman, WA, USA).

### 2.3. Adsorption Determination and Advanced Modeling of MB Dye

The adsorption test was conducted using a stock solution of MB (1 g/L), which was diluted to obtain different concentrations ranging from 50 mg/L to 400 mg/L. Adsorption isotherms associated with the removal of MB by FG adsorbent were conducted at pH 7.0 and temperatures of 25 °C, 40 °C, and 50 °C using 50 mg of FG and 50 mL of MB solution. All MB removal experiments were performed in duplicate, and the results were averaged for data analysis finding standard deviations less than 5.0%. FG–MB suspensions were stirred for 6 h at 150 rpm; then, the liquid phases were separated by means of a centrifugation process. MB dye concentrations were determined with a double-beam UV-visible spectrometer (Shimadzu, UV 1601). The equilibrium adsorption amounts of MB ($q_{e}$) were assessed using the following relationship:

(1) $$q_{e}=\left(C_{0}-C_{e}\right)\frac{\;V}{\;m}$$

where $C_{0}$ (mg/L) is the initial MB concentration, $C_{e}$ (mg/L) is the last MB concentration at equilibrium, *V* (L) is the MB solution volume, and m (g) is the mass of the FG adsorbent.

In this study, three advanced statistical physics models (i.e., monolayer, double-layer, and multilayer) were used as given below.

#### 2.3.1. Monolayer Model (Model 1)

This model suggests that the removed MB molecules can produce a single layer on the FG active sites and the created layer is controlled by the adsorption energy (ΔE). Contrary to the classical Langmuir model, the monolayer model assumes that the functional group of the FG adsorbent can capture an adjustable number of MB molecules [12]. The mathematical expression of Model 1 is presented by Equation (2) [13].

(2) $$q_{e}=\frac{nD_{M}}{1+{\left(\frac{c_{1/2}}{c}\right)}^{n}}\;\;\;\;\;\;\;\;\;\;$$

where *C*_1/2_ means the concentration at half-saturation surface at saturation.

#### 2.3.2. Double-Layer Model (Model 2)

This model assumes the development of two layers of MB molecules directed by different energies (i.e., ΔE1 for the first layer and ΔE2 for the second one). The expression of Model 2 for the removed MB quantity is given by [12].

(3) $$q_{e}=nD_{M}\frac{{\left(\frac{c}{c_{1}}\right)}^{n}+2{\left(\frac{c}{c_{2}}\right)}^{2n}}{1+{\left(\frac{c}{c_{1}}\right)}^{n}+{\left(\frac{c}{c_{2}}\right)}^{2n}}$$

where *c*_1_ and *c*_2_ are the half-saturation concentrations attributed to the first and second layers formed on the FG surface.

#### 2.3.3. Multilayer Model (Model 3)

According to this advanced model, a definite number of removed MB layers can be formed. The total number of removed MB layers (Nt) on the FG active sites is equal to 1 + N2. In addition, it is significant to explain that the fixed number of MB layers can be formed via dye–FG interaction with ΔE1, while the N2 number is linked to the MB–MB interface with ΔE2. The mathematical equations utilized for calculating the physicochemical factors of this advanced model are offered below [12].

(4) $$q_{e}=n\;D_{M}\frac{F_{1}\left(c\right)+F_{2}\left(c\right)+F_{3}\left(c\right)+F_{4}\left(c\right)}{G\left(c\right)}$$

(5) $$F_{1}\left(c\right)=-\frac{2{\left(\frac{c}{c_{1}}\right)}^{2n}}{1-{\left(\frac{c}{c_{1}}\right)}^{n}}+\frac{{\left(\frac{c}{c_{1}}\right)}^{n}\left(1-{\left(\frac{c}{c_{1}}\right)}^{2n}\right)}{{\left(1-{\left(\frac{c}{c_{1}}\right)}^{n}\right)}^{2}},$$

(6) $$F_{2}\left(c\right)=\frac{2{\left(\frac{c}{c_{1}}\right)}^{n}{\left(\frac{c}{c_{2}}\right)}^{n}\left(1-{\left(\frac{c}{c_{2}}\right)}^{n\;N_{2}}\right)}{1-{\left(\frac{c}{c_{2}}\right)}^{n}},$$

(7) $$F_{3}\left(c\right)=-N_{2}\frac{{\left(\frac{c}{c_{1}}\right)}^{n}{\left(\frac{c}{c_{2}}\right)}^{n}{\left(\frac{c}{c_{2}}\right)}^{n\;N_{2}}}{1-{\left(\frac{c}{c_{2}}\right)}^{n}},$$

(8) $$F_{4}\left(c\right)=\frac{{\left(\frac{c}{c_{1}}\right)}^{n}{\left(\frac{c}{c_{2}}\right)}^{2n}\left(1-{\left(\frac{c}{c_{2}}\right)}^{n\;N_{2}}\right)}{{\left(1-{\left(\frac{c}{c_{2}}\right)}^{n}\right)}^{2}},$$

(9) $$G\left(c\right)=\frac{\left(1-{\left(\frac{c}{c_{1}}\right)}^{2n}\right)}{1-{\left(\frac{c}{c_{1}}\right)}^{n}}+\frac{{\left(\frac{c}{c_{1}}\right)}^{n}{\left(\frac{c}{c_{2}}\right)}^{n}\left(1-{\left(\frac{c}{c_{2}}\right)}^{n\;N_{2}}\right)}{{\left(1-{\left(\frac{c}{c_{2}}\right)}^{n}\right)}^{2}}$$

The $R^{2}$ and the root mean square error (RMSE) values were used to select the best advanced statistical model for evaluating the MB adsorption process [12,13].

(10) $$R^{2}=1-\frac{\sum {\left(q_{e,\exp}-q_{e,\mathrm{cal}}\right)}^{2}}{\sum {\left(q_{e,\exp}-q_{e,mean}\right)}^{2}}$$

(11) $$RMSE=\sqrt{\frac{\sum_{i=1}^{m}{\left(Q_{i\;\mathrm{cal}}-Q_{i\;\exp}\right)}^{2}}{m^{\prime }-p}}$$

where $q_{e,\exp}\;\mathrm{and}\;q_{e,\mathrm{cal}}$ refer to the experimental and the calculated MB adsorption capacity (mg/g), respectively. In addition, $m^{\prime }\;$ denotes the experimental datum, and p is the number of changeable factors.

## 3. Results and Discussion

### 3.1. Characterization of FG

**Figure 1:**
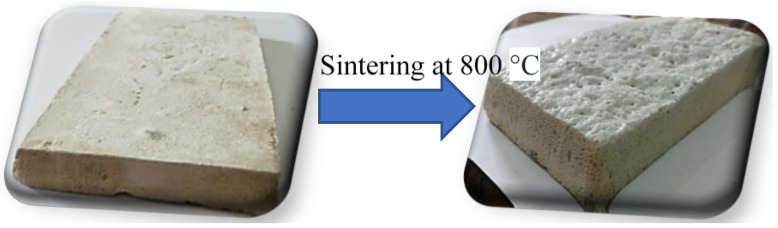
Digital photo of unfoamed and foamed GW (from left to right).

**Figure 2:**
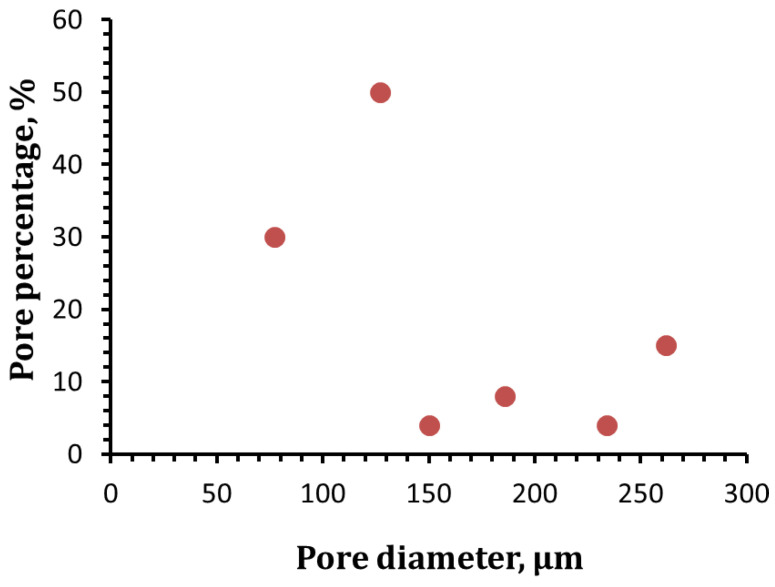
Pore size distribution of FG.

The exposure of NaOH-activated GW to a firing temperature of 800 °C resulted in the formation of a lightweight open-celled structure (Figure 1). Sintering the sodium hydroxide (NaOH) and silicate-rich material (GW) mixture at 800 °C was found to be appropriate in the foaming process. In this activation process, NaOH played a significant role in the acceleration of glass softening as well as the transformation of the amorphous phase to the crystalline one. In addition, the dissolution of the silicate network resulted in the development of a sodium silicate hydrate phase as a main binder in the GW-activated system [32,33]. The heat treatment of alkali-activated GW at 800 °C produced a highly porous system, which resulted from combined water dehydration, gas formation, silica glass transformation, and softening [33,34]. The physicochemical parameters of the tested FG sample exhibited porosity of 91%, bulk density of 0.65 g/cm^3^, compressive strength of 4 MPa, and thermal conductivity of 0.27 W/m·K. As shown in Figure 2, the GF sample had pore diameter ranging from 77 to 262 μm with critical pore diameter of 127 µm.

**Figure 3:**
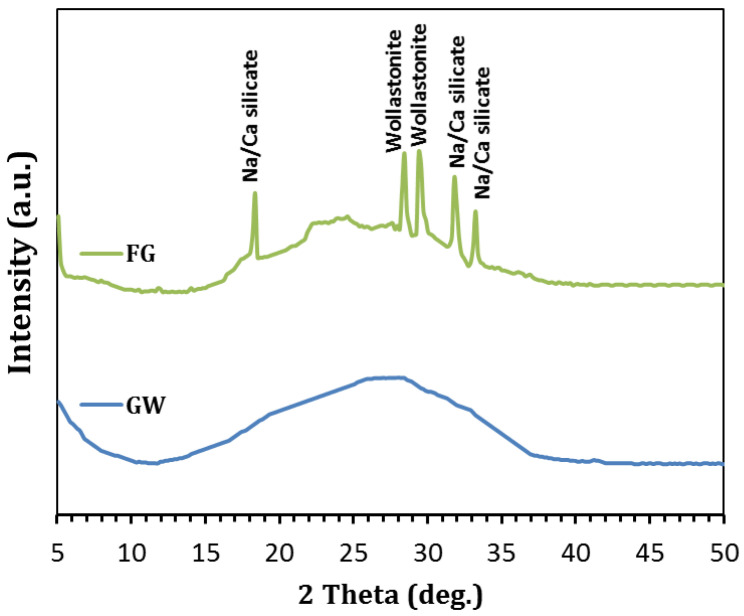
XRD patterns of GW and FG.

**Figure 4:**
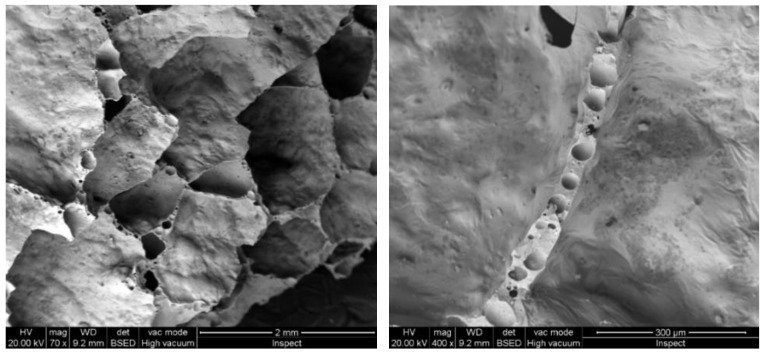
FESEM micrograph of FG.

The XRD patterns of GW and FG are represented in Figure 3. It is clear that GW exhibits a typical amorphous silicate pattern. On the other hand, the exposure of the NaOH-GW mixture to high temperature (800 °C) enhanced the formation of wollastonite and sodium calcium silicate crystalline phases (see Figure 3). The detection of wollastonite and Na/Ca silicate phases reflected the role of NaOH/temperature in the transformation of the glass structure into the crystalline one inside the FG sample [21,22]. Figure 4 displays the FESEM result of the as-synthesized FG. As shown in Figure 4, the bloating process resulted in the formation of pore system with different diameters, suggesting the efficiency of the foamed material in water remediation. In addition, Figure 4 presented interconnected small pores on the wall of each pore inside the FG structure.

**Figure 5:**
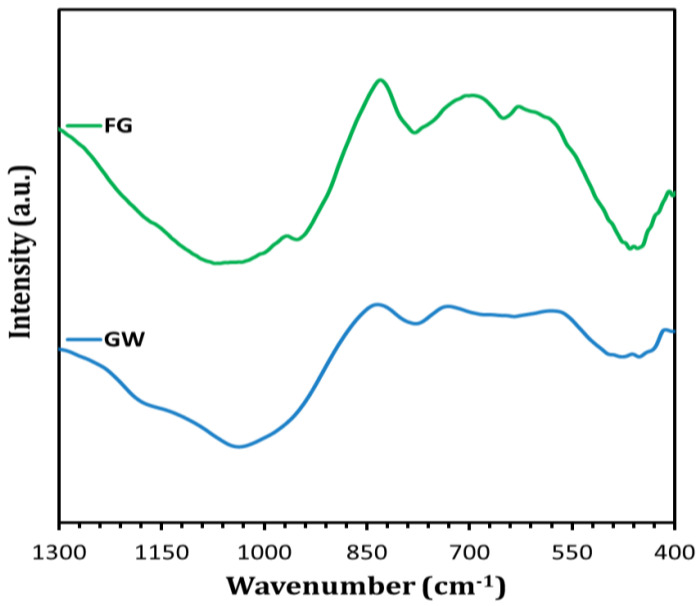
FTIR spectra of GW and FG samples.

The FTIR spectra of GW and FG samples are shown in Figure 5. The absorption bands associated with the bending vibration of Si-O-Si (at 449–776 cm^−1^) and the stretching vibration of Si-O (at 1080 cm^−1^) were observed in GW. The exposure of GW to 800 °C in the presence of NaOH resulted in the formation of a new band related to the stretching vibration of Si-O-Si at lower wavenumber (948 cm^−1^). This could be ascribed to the formation of crystalline wollastonite and sodium calcium silicate phases at elevated temperatures, as proved by XRD analysis (Figure 3).

### 3.2. Theoretical Treatment and Adsorption Mechanism

**Figure 6:**
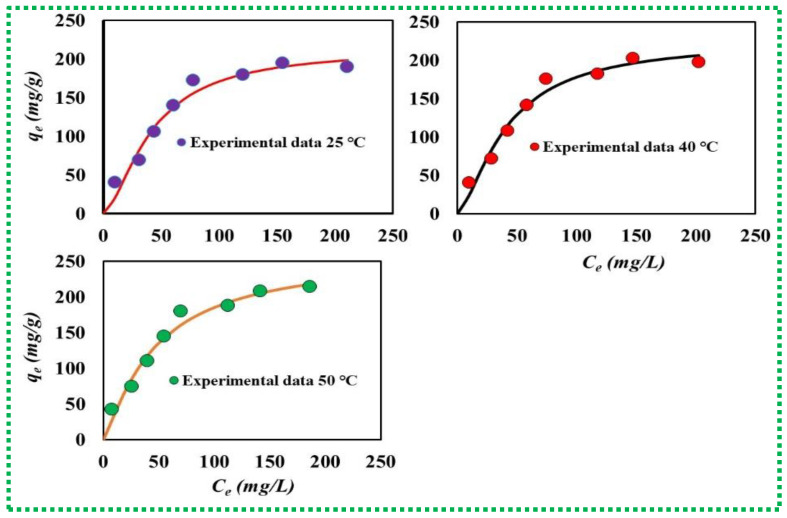
The performance of the monolayer adsorption model in fitting MB uptake by FG adsorbent at 25 °C, 40 °C, and 50 °C.

The modeling results of the three statistical physics models displayed that the values of R^2^ (0.9975–0.9991) and RMSE (7.25–9.14) of Model 1 (monolayer model with one energy) were better than those of the other advanced models (i.e., Model 2 and Model 3), as illustrated in Table 2 and Figure 6. Consequently, the steric (n, D_M_, and Q_sat_) and energetic (ΔE) parameters calculated according to the selected monolayer model with one energy were proven to offer new insights into the interaction between MB molecules and the FG active sites under all tested experimental conditions.

#### Steric Parameter Interpretation

Commonly, the physicochemical parameter of n involved in the advanced adsorption models was used to adjust the suggestion of the Langmuir adsorption model [2,5]. The value of this steric parameter can be =1 (i.e., Langmuir hypothesis), or greater or smaller than unity, which reflects different states of the adsorbent behavior [7,12]. The value of the n parameter at each solution temperature can clearly describe the geometry of the removed MB molecules by the FG active sites. Furthermore, the adsorption mechanism of the MB dye on FG could be either multi-docking or multi-molecular according to the value of this physicochemical parameter. Overall, three cases are used to define the position and mechanism of the captured MB molecules by the FG adsorbent [2,5,7,12].

➣ n < 0.5: Based on this case, two or more adsorption sites of FG can contribute to the uptake of MB molecules, reflecting parallel orientation and a multi-docking mechanism.
➣ 0.5 < n < 1: Under this condition, MB molecules can be concurrently captured by horizontal and vertical positions, where non-parallel orientation is the main geometry if the n value is closer to unity.
➣ n ≥ 1: According to this case, one active site of the FG adsorbent has the ability to remove more than one MB molecule, thus signifying vertical orientation and a multi-molecular mechanism.

**Figure 7:**
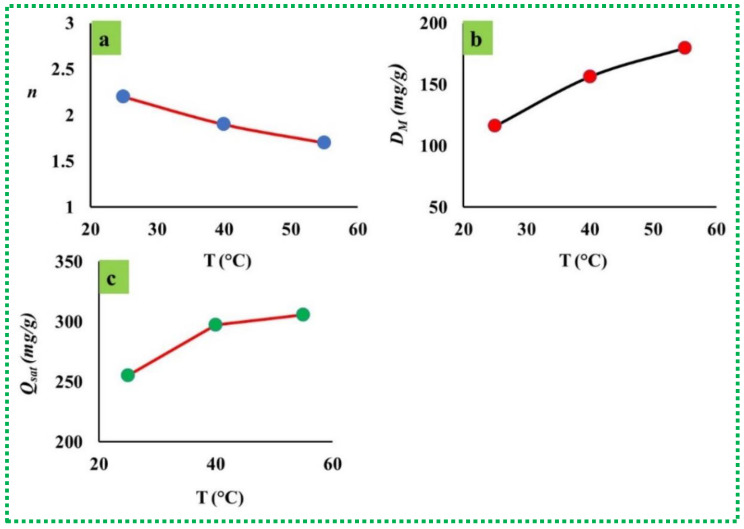
The n, D_M_, and Q_sat_ parameters for the adsorption of MB on FG.

Figure 7 illustrates the behavior of the n parameter at different adsorption temperatures (i.e., 25, 40, and 50 °C), and the equivalent values are also presented in Table 3. The n parameter gave the values of 2.2, 1.90, and 1.70 at 25 °C, 40 °C, and 50 °C, respectively. Therefore, vertical orientation and a multi-molecular mechanism were identified in the MB adsorption process (i.e., the last case, in which n parameter ≥ 1 was achieved) at all temperatures. In addition, it can be concluded that the increment in the solution temperature from 25 to 50 °C resulted in the decrease in the value of the n parameter associated with the adsorption of MB molecules on FG active sites (see Table 3). Therefore, the temperature did not support the binding of MB dye to the adsorption site due to the effect of thermal agitation [12]. This behavior suggests the existence of a specific type of FG active sites that played the main role in the removal process. Furthermore, the aggregation of MB molecules was characterized at all solution temperatures, and this accumulation phenomenon of dye molecules decreased with the increase in the temperature. Consequently, the orientation and mechanism behaviors related to MB adsorption displayed no changes with the increase in the temperature from 25 °C to 50 °C.

The D_M_ steric parameter increased from 115.96 to 179.75 with the increase in the solution temperature from 25 °C to 50 °C (see Figure 7). This result could be linked to the involvement of additional FG active sites in the adsorption of MB molecules. In addition, the increment in the D_M_ value with the increase in the temperature suggested the endothermic performance of the MB-FG adsorption system. In addition, it could be observed that when the D_M_ parameter increased with the temperature, the corresponding value of the n factor decreased (Table 3). Normally, the aggregation of MB molecules (i.e., when the n parameter increased) resulted in the decrease in the occupied FG by the removed dye molecules (i.e., the D_M_ parameter decreased).

Calculating the Q_sat_ parameter is valuable for understanding the adsorption behavior of MB as well as to evaluate the FG removal efficiency. Figure 7 and Table 3 present the performance of Q_sat_ against the temperature. Obviously, the Q_sat_ value of MB dye increased from 255.11 mg/g to 305.58 mg/g with the temperature increase between 25 °C and 50 °C. The increment in the Q_sat_ value with the increase in the temperature reflected an endothermic adsorption process. Increasing the kinetic energies of MB molecules at high temperature resulted in enhancing Q_sat_ at 40 °C and 50 °C. Both the Q_sat_ and D_M_ parameters improved with the temperature, and consequently, the density of FG active sites was recommended to be the principal parameter governing the uptake efficiency of this porous adsorbent.

### 3.3. Energetic Parameter Interpretation

The calculation of the adsorption energy (ΔE) is significant to find a proper interpretation of the interaction between MB molecules and FG active sites. Model 1 suggests the existence of one adsorption energy (ΔE) at each solution temperature that can be involved in the uptake of MB, and this energetic parameter was determined as given below [12].

(12) $$C_{1/2}=C_{s}e^{-\frac{\Delta E}{RT}}$$

where $c_{1/2}\;$ signifies the concentrations at half-saturation and $c_{s}$ refers to MB solubility. R is the ideal gas constant, which is equal to 8.3144621 J/mol K.

Table 3 displays the values of MB adsorption energies at the three solution temperatures. The adsorption energy presented values of 16.75, 17.43, and 17.55 kJ/mol at 25, 40, and 50 °C, respectively (Table 3). Accordingly, ΔE offered positive values at all tested solution temperatures. The positive values of ΔE confirmed the endothermic interaction between the MB molecules and the FG receptor sites. Moreover, the calculated adsorption energies were found to be less than 20 kJ/mol, which suggested that physical interactions (i.e., electrostatic attraction and hydrogen bonding) could have been involved in removing MB molecules by the FG active sites. Furthermore, both of the Q_sat_ and ΔE parameters increased with the temperature (i.e., the two factors had the same trend); therefore, ΔE played an important role in improving the MB adsorption capacity.

### 3.4. Diffusion Mechanism of MB Dye

To estimate the diffusion mechanism of MB into the FG adsorbent, the intra-particle diffusion model was used as given below:

(13) $$q_{t}=k_{p}\;t^{1/2}+C$$

where *k_p_* (mg/g·min) is the rate constant of the intra-particle diffusion model and C (mg/g) is the value of intercept that is attributed to the thickness of the porous medium.

**Figure 8:**
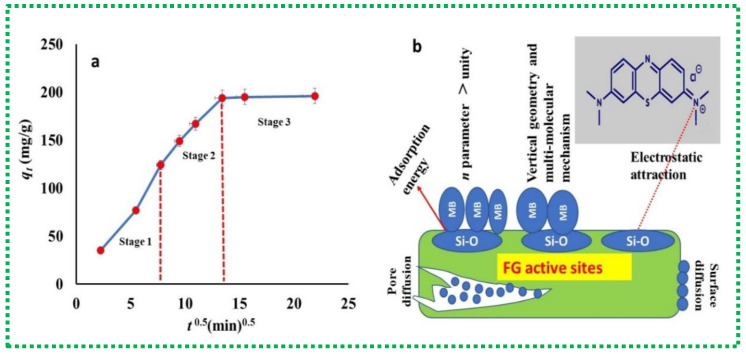
The intra-particle diffusion model (**a**), and the possible interaction mechanism between FG active sites and MB molecules (**b**).

The intra-particle diffusion model was quantified at different adsorption times (5 to 480 min) using the following conditions: 50 mg of FG adsorbent, 50 mL of MB solution (200 mg/L), 25 °C solution temperature, and pH 8.0. The plot of q_t_ against t^1/2^ related to this kinetic adsorption model is illustrated in Figure 8. Based on this model, if the plot is linear and passes through the origin, then the intraparticle diffusion mechanism is the only rate-controlling step [6]. On the other hand, the multi-linear plot of the MB experimental data reflects the presence of different diffusion types. Clearly, this kinetic model distinguishes three different stages in the whole time range of 5 min–480 min (see Figure 8). These dissimilar stages reflect the mass transfer of MB molecules from the solution to the external FG active sites (stage 1), the control of pore diffusion mechanism (stage 2), and the achievement of the equilibrium state (stage 3). The intra-particle diffusion model revealed more than one linear part; therefore, both surface diffusion and intra-particle diffusion mechanisms could be involved in the MB adsorption process [6]. Furthermore, the presence of a pore diffusion mechanism confirmed the porous structure of FG, which played a significant role in the adsorption process.

Overall, the possible interaction mechanism between the negatively charged functional groups of FG and the positively charged MB molecules can be summarized in Figure 8.

### 3.5. Comparison with Other Adsorbents

Table 4 reports the maximum adsorption capacity of MB on FG and the comparison with different raw, modified, and synthetic materials. Obviously, the developed FG presented high MB adsorption capacity as compared with natural (montmorillonite and kaolin), modified (diatomite and rice husk), synthetic porous (Mn-MCM-41 silica composite and zeolite 4A), and magnetic (Fe_3_O_4_/montmorillonite) adsorbents. The presence of active adsorption sites (e.g., Si–O−functional group) and the porous structure of the developed FG were suggested to be the main reasons for the increase in the adsorption capacity of this adsorbent. Based on the listed q_max_ values, the FG adsorbent can be seen as a promising and low-cost porous material for the remediation of MB-containing solutions.

### 3.6. Regeneration of FG Adsorbent

Utilizing an adsorbent several times is considered an important concern in industrial processes for decreasing the water remediation costs [40]. Therefore, to test the stability of the FG adsorbent, the adsorption–desorption round was conducted four times. The FG (50 mg)-loaded adsorbent was thoroughly washed using distilled water and subsequently oven-dried for 24 h at 70 °C. Next, the desorption study was performed using 50 mL of 0.5 M NaOH as an eluent solution at 25 °C. The FG adsorbent loaded with MB molecules was agitated for 6 h at 120 rpm. After all regeneration cycles, the prepared FG presented MB removal percentages (%) of 97, 92, 89, and 83% for cycles 1, 2, 3, and 4, respectively. The decrease in MB adsorption capacity through these cycles can be associated with two probable factors: (I) irreversible adsorption of some amount of the MB dye that could not be desorbed and (II) gradual elimination of porosity and surface chemistry upon processing (i.e., water-driven processes happening during the adsorption cycles and NaOH-driven processes during desorption cycles) [40]. Consequently, the as-synthesized FG adsorbent can be reused many times to remove MB dye without significantly losing in its efficiency and stability. Based on the adsorption/desorption results, FG can be recommended to be a prominent and highly stable adsorbent for the removal of dyes-containing water.

## 4. Conclusions

Foamed glass (FG) was successfully prepared by subjecting a glass waste and NaOH mixture to a treatment temperature of 800 °C. The as-synthesized FG was characterized and applied as an effective adsorbent for the removal of methylene blue (MB) from solutions. Th physicochemical properties (i.e., density, compressive strength, and thermal conductivity) of the fabricated FG were calculated. The heat treatment of alkali-activated GW at 800 °C produced a porous system with a critical pore diameter of 127 µm. Theoretical treatment based on the application of statistical physics models was performed to better understand the MB-FG interface. A monolayer model with one energy site resulted to be the best statistical physics model (i.e., R^2^ ranged from 0.9975 to 0.999, and RMSE ranged from 7.25 to 9.14) in fitting the adsorption data of MB molecules. The calculated steric parameters indicated that vertical orientation and a multi-molecular adsorption mechanism were involved in the MB adsorption process. The density of FG receptor sites improved from 115.96 to 179.75 mg/g with the increase in the temperature, and this parameter played the main role in the adsorption performance of FG. The adsorption capacity increased from 255.11 to 305.58 mg/g with the increase in the temperature, suggesting endothermic interactions between the removed MB molecules and FG active sites. The removal of MB molecules by FG was controlled by physical forces, as the calculated adsorption energies were found to be less than +20 kJ/mol at all solution temperatures. Surface and pore diffusion processes were involved in the MB adsorption system. The FG adsorbent could be easily regenerated and reused four times without a significant decrease in its removal efficiency (i.e., more than 80% of MB removal was kept after four desorption cycles). The current study offers a new strategy for the valorization of silica-rich solid wastes to prepare effective and low-cost adsorbents for organic water contaminants.

## Figures and Tables

**Table 1:** Chemical composition (wt.%) of GW.

Item	SiO_2_	Al_2_O_3_	Fe_2_O_3_	CaO	MgO	SO_3_	Na_2_O	K_2_O	TiO_2_	P_2_O_5_	Cl-	LOI	Total
GW	80.01	0.93	0.20	6.93	3.53	0.26	7.45	0.13	0.09	0.02	0.02	0.31	99.98

**Table 2:** Results of MB adsorption isotherms using the utilized statistical physics models.

	Temperature					
	25 °C		40 °C		50 °C	
	R^2^	RMSE	R^2^	RMSE	R^2^	RMSE
Model 1	0.9975	9.14	0.9989	8.08	0.9991	7.25
Model 2	0.9715	13.89	0.9808	10.15	0.9908	8.05
Model 3	0.9804	11.07	0.9706	11.73	0.9897	9.43

**Table 3:** Steric and energetic parameters for MB adsorption on FG.

T (°C)	n	$D_{M}(\mathrm{mg}/g)$	$Q_{sat}(\mathrm{mg}/g)$	ΔE(kJ/mol)
25	2.20	115.96	255.11	16.75
40	1.90	156.36	297.08	17.43
50	1.70	179.75	305.58	17.55

**Table 4:** Comparison of adsorption capacity of different materials and the developed FG.

Dye	Adsorbent	Qmax (mg/g)	Reference
MB	Montmorillonite clay	64	[34]
MB	Fe_3_O_4_/montmorillonite	106	[34]
MB	Fibrous clays	39–85	[35]
MB	Mn-composite mesoporous MCM-41 silica	132	[36]
MB	Modified diatomite	105	[37]
MB	Chitosan/magnetic composite	201	[38]
MB	Kaolin	45	[39]
MB	Zeolite 4A	22	[39]
MB	Ball clay	25	[39]
MB	Graphene	153	[39]
MB	Modified rice husk	65	[39]
MB	FG	255.11	Current study

## Data Availability

Data are contained within the article.
